# *Gelsemium elegans* Poisoning: A Case with 8 Months of Follow-up and Review of the Literature

**DOI:** 10.3389/fneur.2017.00204

**Published:** 2017-05-17

**Authors:** Zhou Zhou, Lei Wu, Yuhua Zhong, Xiaobo Fang, Yanmei Liu, Hongbing Chen, Weixi Zhang

**Affiliations:** ^1^Department of Neurology, The First Affiliated Hospital, Sun Yat-Sen University, Guangzhou, China

**Keywords:** coma, *Gelsemium elegans*, poisoning, depressive disorder, suicide

## Abstract

**Background:**

*Gelsemium elegans* (*G. elegans*) is a toxic plant indigenous to Southeast Asia. It is highly poisonous due to its strong respiratory depressive effect. However, *G. elegans* poisoning cases have not been summarized comprehensively and are rarely reported in English journals. Furthermore, none of the present reports present prognosis in detail.

**Case presentation:**

A 26-year-old female was found comatose at home and brought to the hospital with deep coma, hypoxia, and acidosis. After mechanical ventilation for hours, the patient recovered from coma with sequelae of impaired short-term memory, disorientation, and childish behaviors. Brain magnetic resonance imaging (MRI) showed bilateral hippocampus and basal ganglia damage due to hypoxia. During 8 months of follow-up, both her symptoms and brain MRI scan improved significantly.

**Conclusion:**

*G. elegans* is highly toxic. Although patients may die within 30 min due to its strong respiratory depressive effect, they can survive with timely respiratory support and enjoy gradual improvement without delayed postanoxic encephalopathy.

## Background

*Gelsemium elegans* (*G. elegans*) is one of three species of *Gelsemium*, a genus of flowering plants in the Gelsemiaceae family. It is indigenous to Southeast Asia and can be specifically found in southeast China, India, Indonesia, Laos, Malaysia, north Myanmar, north Thailand, and Vietnam. *G. elegans* is an evergreen shrub, with long stems up to 12 m, and grows as a twining vine, interweaving with other surrounding vegetation. It may interweave with other edible plants or be mistaken for various lookalike therapeutic herbs, leading to inadvertent consumption and poisoning. *G. elegans* is highly poisonous due to its strong neurological and respiratory depressive effects (1). Oral administration of crude extracts of *G. elegans* at doses of 10, 15, 20, and 25 mg/kg caused death in 11, 50, 72, and 100%, respectively, of test mice (2). The high concentration of alkaloids appears to be responsible for the toxic effects of the plant. To date, a total of 121 alkaloids have been found in *Gelsemium*, and gelsenicine is the most toxic alkaloid in *G. elegans* (LD_50_ ~0.128 mg/kg mice, intraperitoneally; 0.26 mg/kg rat, intraperitoneally; and 0.15 mg/kg rat, intravenously), whereas koumine is the most abundant alkaloid and exhibits mild toxicity (LD_50_ ~100 mg/kg mice, intraperitoneally) (3). In contrast to an intensive study of *G. elegans* on phytochemistry, *G. elegans* poisoning cases have not been summarized comprehensively and are rarely reported in English journals. Furthermore, none of the present reports presents prognosis in detail. Here, we report a case of *G. elegans* poisoning.

## Case Presentation

The patient, an educated 26-year-old Teochew woman, was found unconscious in her bedroom around midnight. She was immediately sent to the nearest emergency room. At arrival, she had a Glasgow coma scale score 3/15, respiratory rate 36 breaths/min, pupils 6 mm in diameter without light reaction, heart rate 112 beats/min, blood pressure 152/100 mmHg, and SpO_2_ 36%. Intubation was carried out immediately, and she was placed on mechanical ventilation; half an hour later, arterial blood gas analysis revealed pH 7.22, PaO_2_ 35 mmHg, PaCO_2_ 57.5 mmHg, SO_2_ 55%, lactate 5.0 mmol/l, HCO_3_ 22.8 mmol/l, SB −3.8 mmol/l, and AB −5.1 mmol/l. She regained consciousness and was weaned from mechanical ventilation with normal arterial blood gas analysis results 6 h later. After the first aid, her vital signs were normal, and her condition did not fluctuate or deteriorate. She continued to be hospitalized at her local medical center for 11 days but failed to identify the cause of the coma. To find out the cause of the coma, she was sent to our hospital.

**Figure 1:**
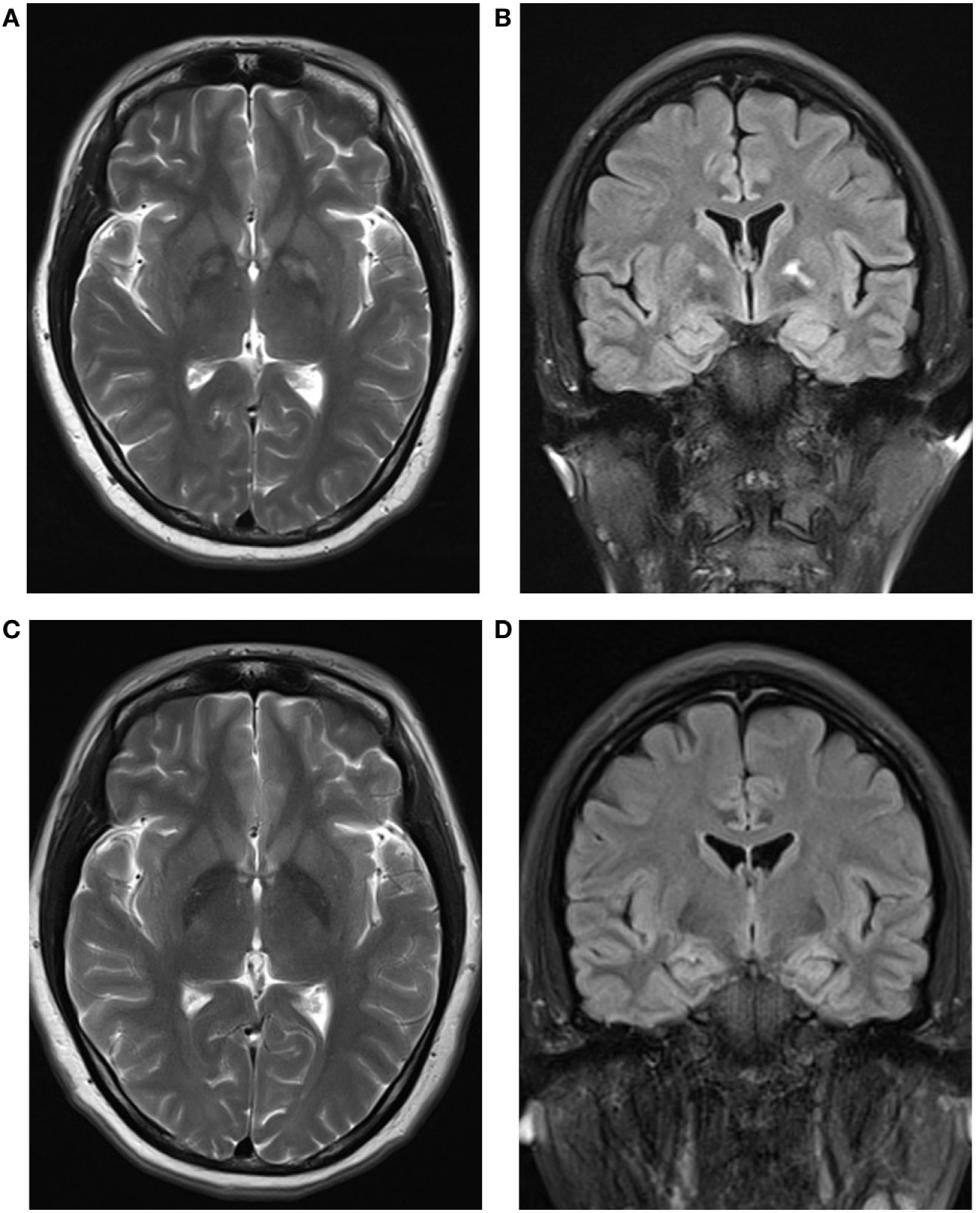
**(A,C)** Axial T2-weighted magnetic resonance imaging (MRI) slices showing symmetrical hyperintensity in the globus pallidus at 17 days **(A)** and 3.5 months **(C)** after poisoning, demonstrating a reduction in globus pallidus hyperintensity. **(B,D)** Coronal fluid-attenuated inversion recovery MRI slices at 17 days **(B)** showing asymmetry of the hippocampus larger on the left side and improved symmetry at 3.5 months **(D)** after poisoning.

At arrival in our department, she presented with euphoria and childish behaviors. She was unable to recall how she sank into coma. Her past medical history was unremarkable. Physical examination was only notable for disorientation to time and place and impaired short-term memory. Results of routine blood, serum, urine, and stool tests and cerebrospinal fluid analysis were unremarkable. The Mini-Mental State Examination (MMSE) score was 23/30. Brain computed tomography scan was normal. Cerebral magnetic resonance imaging (MRI) showed abnormal signals from bilateral globus pallidus and asymmetry of the hippocampus larger on the left side (Figure 1). Magnetic resonance spectroscopy (MRS) showed that the values of NAA/(Cho + Cr) at bilateral hippocampus were less than normal in both sides but more prominent in the left.

History was taken in detail several times; finally, her husband recalled that there was a bottle of broth of herbs at her bedside table. The herbs looked like *G. elegans*. Samples of interest taken in the scene were then analyzed and toxic *Gelsemium* alkaloids were detected by the China National Analytical Center of the Chinese Academy of Sciences. Therefore, diagnosis of *G. elegans* poisoning was established.

Gradually, euphoria and childish behaviors wore off within 1 month. However, she presented with depressed mood and did not respond when asked whether she ingested the broth of *G. elegans*. She was discharged on the 36th day after onset. On the first follow-up, 3.5 months after intoxication, we evaluated her with Hamilton Depression Rating Scale (24 items), and the score was 23. During this evaluation, the patient informed us that she ingested the broth of *G. elegans* the evening of initial presentation, and she knew it was toxic. An antidepressant (escitalopram) was added. The MMSE score at that time was 26/30, with short-term memory improved, but the orientation ability to time and place was still impaired. MRI showed that the range of abnormal signals of bilateral globus pallidus was reduced (Figure 1). The symmetry of the hippocampus was also improved in the MRI scan (Figure 1), but MRS still showed NAA/(Cho + Cr) < 0.75. On the second follow-up, 8 months after intoxication, our patient was able to work, although she felt much more challenge than before. The score of Hamilton Depression Rating Scale (24 items) declined to 19. The MMSE score was 28/30, with mild impairment of orientation ability to time and place. We added the Montreal Cognitive Assessment this time, which detected short-term memory impairment and disorientation more sensitively of our patient, and the score was 25/30. MRI and MRS were quite similar to that of the first follow-up.

## Discussion

Reports of *G. elegans* poisoning in English are rare. To our knowledge, there are only four papers that involved the subject of *G. elegans* poisoning (summarized in Table 1) (1, 4–6). However, using the Chinese name of *G. elegans* as the search keyword, we found another 28 papers (7–34) that reported *G. elegans* poisoning cases in Wanfang Data, a major Chinese academic paper database of China. Most of these cases are from collective food poisoning accidents due to mistaking *G. elegans* as non-toxic herbs (7, 8, 11–13, 15, 16, 22, 23, 25–29). The most common reason for *G. elegans* poisoning is mistaken ingestion, as its morphology is similar to some non-toxic Chinese medicinal herbs (1, 6). The second most common reason is suicide (10, 12, 14, 17, 18). Some other rare causes include homicide (19) and using *G. elegans* to treat disease without caution (14, 30).

**Table 1:** **Summary of previous English case reports of *Gelsemium elegans* poisoning**.

Year	Patient number	Age	Reason	Clinical manifestation	Death time
1908	1	50	Homicide	Pain, muscles twitching and contracting	3.5 h
	2	Unknown	Suicide	Pain, dazed, semiconsciousness, dilated pupils insensible to light, slight contraction of the muscles, especially of the upper limbs	Survived
1988	1	67	Suicide	Unknown	4 h
2007	1	65	Ingestion by mistake	Dizziness, generalized weakness, and nausea followed quickly by unconsciousness, bradypnea, dilated pupils insensible to light	Survived
	2	69	Ingestion by mistake	Dizziness, generalized weakness, vomiting, tachycardia	Survived
2016	1–11	28–58	Ingestion by mistake	Dizziness, blurred vision, barylalia, limb weakness, convulsions, dyspnea, coma, blepharoptosis, diplopia, vomiting, bradycardia, tachypnea, tachycardia, shallow, and irregular breathing, reduced body temperature, decreased blood pressure, respiratory failure	5 deaths (1 died within 1 h) and 6 survivors

The clinical features of *G. elegans* poisoning can be summarized as follows. First, the incubation period is very short, with onset of symptoms mostly occurring within 20 min after ingestion (27). The shortest onset of symptoms after ingestion recorded is 5 min (28) and the longest is 2 h (29). In addition, the external application of *G. elegans* to treat skin disease can also be harmful, with an incubation period of less than 1 h (30). Second, in most cases, the initial manifestations of *G. elegans* poisoning are gastrointestinal abnormalities followed by neurological abnormalities and respiratory depression. However, it is indeed multifarious (Table 2 lists the common manifestations being mentioned in the literature) and may vary according to the dose of ingestion. In those who ingest higher doses, neurological abnormalities and respiratory failure will be more prominent and earlier to present (14) and even cover up gastrointestinal manifestations. Third, among the multifarious clinical manifestations of *G. elegans* poisoning, respiratory depression is the most prominent and the main cause of death. Patients may die within 30 min (13) to 2.5 h (27) due to respiratory failure. Although the chemical composition of *G. elegans* is complex, the alkaloids appear to be responsible for the toxic effects of the plant (3). Animal studies demonstrate that many alkaloids possess respiratory depressive effects (2, 35), such as gelsemicine, sempervirine, koumicine, koumine, kouminicine, and kounidine. Fourth, *G. elegans* poisoning can also lead to the dysfunction of the heart, kidneys, and/or liver, but they are uncommon. Long QT syndrome, torsade de pointes, and third-degree atrioventricular block are reported (9, 14, 16, 21, 26, 32). The overall incidence of arrhythmia is less than 10%. Given the fact that cardiovascular disorders appear after respiratory arrest, it seems the alkaloids of *G. elegans* affect the cardiovascular system at a lesser extent, which differs from aconitine or daturine intoxication (16). It is also proposed that *G. elegans* ingestion may lead to direct renal injury, manifesting as oliguria, anuria, and progressive elevation of blood urea nitrogen and creatinine on the second or third day of intoxication (28). Hepatic function may be affected as well: for about 14% patients, jaundice and/or elevation of alanine transaminase and/or aspartate transaminase may occur several days after intoxication (28).

**Table 2:** **Common manifestations being mentioned in the literature**.

System	Manifestation
Digestive system	Burning throat, nausea, vomiting, abdominal pain, abdominal distension
Nervous system	Dizziness, vertigo, dysarthria, dysphagia, blurred vision, diplopia, drooping eyelids, mydriasis, miosis, absence of pupillary light reflex, gait ataxia, fatigue, convulsion, numbness, restlessness, loss of consciousness
Respiratory system	Chest tightness, tachypnea, bradypnea, shortness of breath, irregular breathing, respiratory arrest, cyanosis
Circulatory system	Palpitation, bradycardia, tachycardia, cardiac arrest, decrease of blood pressure

The diagnostic process for *G. elegans* intoxication may be time consuming and could likely involve forensic investigation (10). *Gelsemium* alkaloids can be detected in the urine, suggesting that urinary gelsemine is a practical marker of *Gelsemium* exposure in human subjects (36). Because of the relatively short half-life of *Gelsemium* alkaloids (37), urine specimens need to be collected in a timely manner.

Gastric lavage, activated charcoal, and cathartics are all used for gastrointestinal decontamination in the treatment of *G. elegans* poisoning. The large majority of the reported literature considers gastrointestinal decontamination as the main focus of treatment (12, 17, 19, 21, 33). However, more recent studies have pointed out that there is an increased risk of aspiration associated with these practices. Sudden respiratory arrest may occur during gastrointestinal decontamination procedures, which cannot be treated easily in time to rescue the patient successfully. Therefore, a protected airway, either when the patient is alert with intact airway reflexes or under intubation, is essential prior to the initiation of any such procedure. In reality, however, owing to the very rapid absorption of *Gelsemium* alkaloids from the gastrointestinal tract, the value of gastrointestinal decontamination is in itself questionable. As stated earlier, patients are likely to die within 1 h (1) or even 30 min (13) due to respiratory arrest. Therefore, we believe that the rule of treatment is to closely monitor respiration and to provide respiratory support as soon as possible when necessary. To enhance poison elimination, many authors adopt hemodialysis as an effective therapy (21, 22, 33), although it is not well proven or documented in this particular context. The safety of hemodialysis is non-controversial among authors and the likelihood of liver or kidney injury as an effect of *Gelsemium* poisoning justifies its application (28). In our case, the major intervention was timely respiratory support. After the acute phase, we tried hyperbaric oxygen therapy (HBOT) on the advice of HBOT experts. However, this therapy lacked satisfactory evidence to begin with and, perhaps unsurprisingly, failed to produce significant effects.

There is scant information regarding the prognosis of *G. elegans* poisoning in previous case reports; these either summarize the case as “recovered and discharged” (7) or “became seriously ill and experienced no symptom relief after medical treatment” (1). The fact is that nearly all cases are reported by emergency-room doctors who are quite unlikely to reevaluate these patients. As mentioned earlier, our patient gradually returned to baseline with some residual dysfunction (e.g., minimal short-term memory impairment and disorientation). There is a need for more observations to define the prognosis of *G. elegans* poisoning.

## Conclusion

*Gelsemium elegans* is highly toxic. Although patients may die within 30 min due to its strong respiratory depressive effect, they can survive with timely respiratory support and enjoy gradual improvement without delayed postanoxic encephalopathy.

## Ethics Statement

No investigation or intervention was performed outside routine clinical care for this patient. As this is a case report, without experimental intervention into routine care, no formal research ethics approval is required. Written, fully informed consent was given and recorded from the patient.

## Author Contributions

ZZ, LW, YL, HC, and WZ were involved in the workup of the patient, planning and conducting investigations, and providing clinical care. They reviewed and revised the manuscript and approved the final manuscript as submitted. ZZ, YZ, XF, and WZ planned the case report, drafted the initial manuscript, reviewed and revised the manuscript, and approved the final manuscript as submitted.

## Conflict of Interest Statement

The authors declare that the research was conducted in the absence of any commercial or financial relationships that could be construed as a potential conflict of interest. The reviewers, SW and MR, handling editor declared their shared affiliation, and the handling editor states that the process nevertheless met the standards of a fair and objective review.
